# Acute effects of LSD on amygdala activity during processing of fearful stimuli in healthy subjects

**DOI:** 10.1038/tp.2017.54

**Published:** 2017-04-04

**Authors:** F Mueller, C Lenz, P C Dolder, S Harder, Y Schmid, U E Lang, M E Liechti, S Borgwardt

**Affiliations:** 1 Department of Psychiatry, Universitäre Psychiatrische Kliniken, University of Basel, Basel, Switzerland; 2 Division of Clinical Pharmacology and Toxicology, Department of Biomedicine and Clinical Research, University Hospital Basel, Basel, Switzerland

## Abstract

Lysergic acid diethylamide (LSD) induces profound changes in various mental domains, including perception, self-awareness and emotions. We used functional magnetic resonance imaging (fMRI) to investigate the acute effects of LSD on the neural substrate of emotional processing in humans. Using a double-blind, randomised, cross-over study design, placebo or 100 μg LSD were orally administered to 20 healthy subjects before the fMRI scan, taking into account the subjective and pharmacological peak effects of LSD. The plasma levels of LSD were determined immediately before and after the scan. The study (including the *a priori*-defined study end point) was registered at ClinicalTrials.gov before study start (NCT02308969). The administration of LSD reduced reactivity of the left amygdala and the right medial prefrontal cortex relative to placebo during the presentation of fearful faces (*P*<0.05, family-wise error). Notably, there was a significant negative correlation between LSD-induced amygdala response to fearful stimuli and the LSD-induced subjective drug effects (*P*<0.05). These data suggest that acute administration of LSD modulates the engagement of brain regions that mediate emotional processing.

## Introduction

Lysergic acid diethylamide (LSD), a potent psychoactive substance,^1^ induces profound changes in various mental domains, including perception, self-awareness and emotions.^2, 3^ As with the other psychedelics (for example, psilocybin and mescaline), these effects are mainly mediated through agonism at the serotonin 5-HT_2A_ receptor.^1, 4^ Currently, there are renewed efforts to use substances like LSD and psilocybin in basic research and clinical practice.^2, 3, 5, 6, 7^ Psilocybin has been studied as a treatment option for addiction, depression and for anxiety in patients with advanced stage cancer.^8, 9, 10, 11^ LSD has been shown to reduce anxiety in patients with life-threatening diseases.^12^ With the investigation of its basic pharmacological and psychological effects, there is also rising interest in the neuronal correlates of the LSD-induced altered state of consciousness. Although several modern studies on psilocybin have been conducted, recent data on LSD in humans are still very limited.^1^

Functional neuroimaging provides a sensitive means of examining how LSD acts on the brain. No data investigating LSD effects on emotion processing have yet been published. The aim of the present study was therefore to investigate these acute effects of LSD using functional magnetic resonance imaging (fMRI). Using a double-blind, randomised, cross-over study design, placebo or 100 μg LSD were orally administered to 20 physically and mentally healthy participants 2.5 h before the fMRI scan, taking into account the subjective and pharmacological peak effects of LSD.^2, 6^ Subjects only had minimal lifetime exposure to illicit drugs; notably, only two subjects had had prior experience with a psychedelic, both on one occasion only. During the fMRI scan, human fearful and neutral faces of a well-validated paradigm were presented. To test our hypothesis that there were differences between placebo and LSD during processing of emotional stimuli, trials for fearful faces were contrasted against trials for neutral faces. We thereby focused on the amygdala as one central part of neural emotion processing, in particular, of anxiety^13, 14^ and additionally included two other regions (the fusiform gyrus and the medial frontal gyrus) known to be responsive to fearful faces.^14^ Differences between placebo and LSD conditions were evaluated by second-level paired *t*-test analysis. In addition, the amygdala response to fearful faces after LSD was correlated with the subjective drug effect, as assessed by a visual analogue scale directly before the scan. The primary and *a priori*-defined study hypothesis was that LSD would decrease the amygdala response to fearful stimuli and that this decrease would be associated with the subjective psychedelic effects.

## Materials and methods

We used a randomised, placebo-controlled, double-blind, cross-over design. Each participant completed two study sessions, with a washout period of at least 7 days between the sessions. The study was approved by the Ethics Committee for Northwest/Central Switzerland (EKNZ) and by the Federal Office of Public Health. Written informed consent was obtained from all the participants. The study (including the *a priori*-defined study end point) was registered at clinicaltrials.gov before study start (NCT02308969).

### Subjects

The subjects were recruited by advertisement and word of mouth. The sample size was determined by power analysis based on previous data.^15, 16^ The exclusion criteria were age <25 or >65 years, pregnancy (as determined by urine test), nursing, hypertension (>140/90 mm Hg) or hypotension (systolic blood pressure<85 mm Hg), cardiac or neurological disorders, use of any regular medication, as determined by medical history and general medical examination including electrocardiography, blood chemistry and haematology, use of illicit drugs (except cannabis) >10 times or any time within the previous 2 months (as assessed by the history and urine test for benzodiazepines, cocaine, amphetamines, methadone, opiates and barbiturates), smoking of >10 cigarettes per day, history of drug dependence, personal or first-degree relative with a history of seizures, personal or first-degree relative with an axis I major psychiatric disorder (as determined by general medical history and a semi-structured interview for Diagnostic and Statistical Manual of Mental Disorders, fourth edition). The subjects provided written informed consent and received monetary compensation for their participation.

### Study procedure

The study included a screening visit, two 25 h test sessions and an end of study visit. The experimental sessions took place in a quiet room in the University Hospital of Basel, Switzerland. The study dates were between December 2014 (first subject screened) and September 2015 (last end of study session). The participants were monitored for adverse reactions and events during the study dates and at the end of study visit. All the adverse events were recorded. The participants were instructed to abstain from any illicit drugs during the whole study period and, additionally, to abstain from caffeine, chocolate and alcohol for at least 8 h before the sessions. The urine drug tests (for tetrahydrocannabinol, benzodiazepines, cocaine, amphetamines, methadone, opiates and barbiturates) were taken randomly on one of the two sessions. In women, pregnancy tests were performed before every session. Except for tetrahydrocannabinol, which can be detected for several weeks, detection of any drug of abuse resulted in study exclusion. A light standardised breakfast was served at both the sessions. Placebo and LSD were administered orally, 2.5 h before the MRI scan at 0900 h, taking into account the subjective and pharmacological peak effects of LSD.^2, 6^

### Drugs and randomization

Gelatin capsules containing 100 μg d-lysergic acid diethylamide hydrate (Lipomed, Arlesheim, Switzerland) and identical capsules containing mannitol were prepared. Each subject received either placebo or LSD on two study sessions in a counterbalanced manner. Only the person dispensing the substance (who was not further involved in conducting the study) was aware of the treatment assignment. Subjects and study personnel were blind to the treatment order.

### Image acquisition

Scanning was conducted on a 3 Tesla MRI system (Magnetom Prisma, Siemens Healthcare, Erlangen, Germany), using a 20-channel phased array radio frequency head coil. Functional MRI acquisition was based on an interleaved T2*-weighted echo planar imaging sequence, with 39 axial slices with a slice thickness of 3 mm, a 0.5 mm inter-slice gap, a field-of-view of 228 × 228 cm^2^ and an in-plane image matrix size of 76 × 76—resulting in 3 × 3 × 3 mm^3^ resolution. The corresponding repetition time was 2.5 s, echo time 30 ms and bandwidth=2350 Hz per pixel. In total, 152 volumes were acquired (including three dummy scan volumes to ensure signal stabilization).

### Subjective effect measurements

The visual analogue scale ‘Any subjective drug effects' was used to assess the overall subjective response to LSD before the scan. The visual analogue scale was presented as a 100 mm horizontal line (0–100%) marked ‘not at all' on the left and ‘extreme' on the right. The scale was rated by the volunteers 2 h after the administration of LSD or placebo.

### Plasma levels

The blood was collected into lithium heparin tubes 2 and 3 h after the administration of LSD and placebo, respectively. The blood samples were immediately centrifuged and rapidly stored at −20 °C until analysis. LSD concentrations in plasma were determined using a validated liquid chromatography-tandem mass spectrometry method.^6^

### fMRI paradigm

During the fMRI acquisition, the study subjects participated in a 6 min experiment based on event-related design implemented with E-Prime 2.0 (Psychology Software Tools, Pittsburgh, PA, USA). During the task, participants were presented with 10 different facial identities (pictures of human faces from the Ekman & Friesen series of Pictures of Facial Affect), each expressing 50 or 100% intensities of fear or a neutral expression. There were thus 30 different facial stimuli in total. Each face was shown twice for 2 s, resulting in a total of 60 stimuli during the paradigm. The order of facial identities and expression type was pseudo-randomised to prevent successive presentation of the same identity or facial expression type. The length of the interstimulus interval, during which subjects viewed a fixation cross, was varied from 3 to 8 s according to a Poisson distribution, with an average interval of 5.9 s. To ensure maximal attention to the presented faces, subjects were requested to decide on the gender of face stimuli by pressing a left or a right button. Accuracy and reaction times were monitored and recorded.

### Data analysis

The data analysis was performed using SPM12 (http://www.fil.ion.ucl.ac.uk/spm/). All the volumes were slice time corrected, realigned to the first volume, co-registered to the pre-processed T_1_-weighted structural volume, normalized into a standard stereotactic space (Montreal Neurological Institute, MNI) and smoothed with a 6 mm full width at half maximum Gaussian kernel. The dummy scans were excluded from any further processing and the remaining volumes were quality checked for severe head motion and image artefacts. the subjects with head motion of >2 mm translation or >2° rotation were excluded. During model specification, the onset times for each trial of neutral, 50% and 100% fearful faces were convolved with a canonical haemodynamic response function. The serial correlations were removed with a first-order autoregressive model and a high-pass filter (128 s) was applied to remove low frequency noise. The six motion parameters for translation and rotation were entered as nuisance covariates. In addition, time and dispersion derivatives were included in the individual design matrix during the first-level analysis. Each trial for 50 and 100% fearful faces was then contrasted against neutral faces, and then produced a subject-specific contrast image propagated to the second-level analysis. One-sample *t*-tests were used to assess the activity induced by the main effect task over all included subjects. The threshold over the whole brain was set at *P*=0.05, corrected for multiple comparisons (family-wise error, FWE). Differences between the LSD and placebo treatment were evaluated by a second-level paired *t*-test. Whole-brain threshold was set at *P*=0.001, uncorrected for multiple comparisons, with an extent threshold of *k*=10 voxels. We restricted our analysis to three meta-analytically identified^14^ regions of interest, namely the amygdala, the fusiform gyrus and the medial frontal gyrus. Those regions were specifically described to be involved in the processing of fearful faces compared with neutral faces.^14^ Based on the Harvard-Oxford Atlas for cortical and subcortical structures, a mask comprising those regions was created. Small volume correction was used for clusters observed within this hypothesized region of interest. The statistical threshold was adjusted to provide a FWE of *P*<0.05, corrected for small volumes. The small volume correction was performed in the global maximum, with a sphere of 5 mm, in accordance with previous fMRI studies on amygdala activity.^17, 18^

The correlation with the subjective effect of LSD in the visual analogue scale was performed using the extracted beta values of the amygdala cluster under the LSD condition. We thereby used the ‘100% fearful versus neutral contrast' to obtain the distinct effect of the fearful stimuli. The calculations were performed using SPSS version 23.00 (IBM, Zurich, Switzerland).

## Results

We included data sets from 20 healthy subjects—9 men, 11 women; mean age 32±10.2 years; range: 25–58 years, all right-handed and all but one with an academic background, originally with 24 study participants. The data sets from four subjects were excluded because of artefacts due to head movements. The lifetime drug use of the 20 included subjects is shown in Table 1. None of the participants tested positive for any drug (including tetrahydrocannabinol) in the screening or test session. No serious adverse reactions or events occurred during the whole period of the study in any of the participants. The plasma levels of LSD were determined immediately before and after the scan and were 1.3±0.6 ng ml^−1^ (mean±s.d.) and 1.1±0.5 ng ml^−1^ (mean±s.d.), respectively.

### Task performance

The differences between the LSD and placebo conditions in task performance were assessed using paired *t*-tests. The mean subject response times did not differ significantly between the two conditions (LSD: 964±128 ms (mean±s.d.); placebo: 910±289 ms (mean±s.d.); *t*_21_=2.0, *P*=0.06). Furthermore, no significant differences were found between the conditions in correctness of response (LSD: 93.1±10.8% (mean±s.d.); placebo: 97.3±3.3% (mean±s.d.); *t*_21_=−1.8, *P*=0.08) or absence of button presses (LSD: 4.5±9.3% (mean±s.d.); placebo: 1.3±1.8% (mean±s.d.); *t*_21_=1.5, *P*=0.16).

### Effect of task

With both treatments (*n*=40), viewing neutral faces versus baseline was associated with bilateral activation in a network comprising the cerebellum, fusiform gyrus, occipital gyrus and the middle cingulate gyrus and lateral activation in the left frontal and lingual gyrus (FWE-corrected at *P*<0.05).

Viewing 100 and 50% fearful faces versus baseline was associated with bilateral activation in the cerebellum, fusiform gyrus, occipital gyrus, middle superior parietal lobule and lateral activation in the left cingulate and frontal gyrus (FWE-corrected at *P*<0.05). Under the placebo condition, presentation of fearful faces induced a significant (small volume correction, *P*<0.05 FWE cluster level) activation of the left amygdala (MNI_max_ *x*=−20, *y*=−12, *z*=−12; cluster size 22; *Z*-score 3.59) compared with presentation of neutral faces.

### Effect of LSD on neural response to fearful versus neutral faces

**Figure 1:**
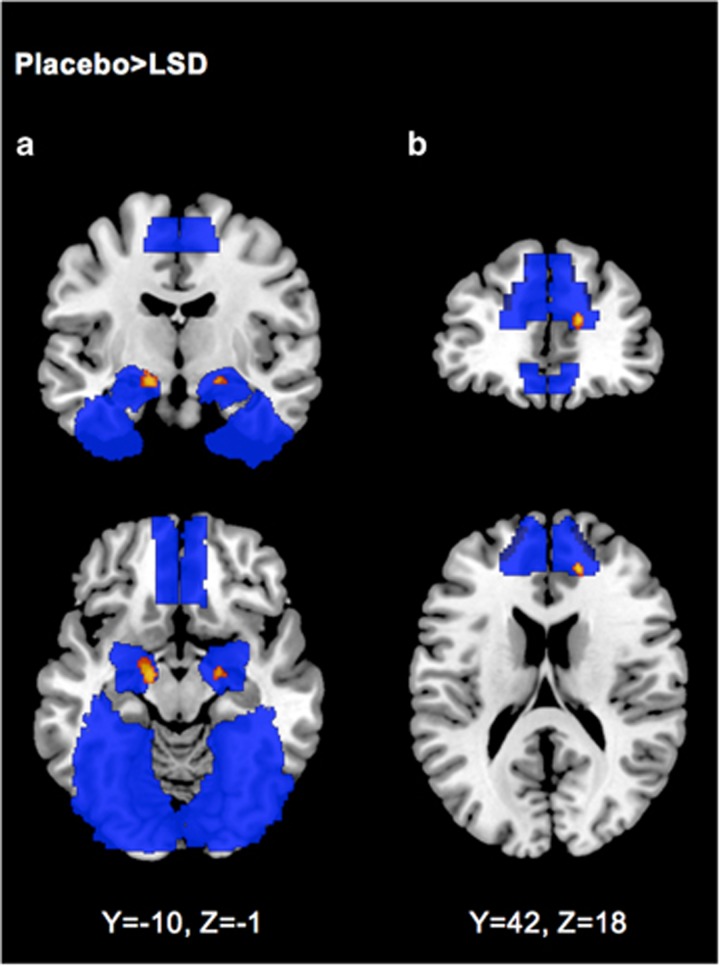
Neural response to fearful versus neutral faces after placebo compared with LSD treatment. LSD decreased reactivity (shown in red-yellow) to fearful faces in the amygdala (**a**) and the right medial frontal gyrus (**b**). Regions of interest (amygdala, fusiform gyrus, medial frontal gyrus) are shown in blue. Threshold *P*<0.001, *k*=10. Right is right side of the brain. LSD, lysergic acid diethylamide.

**Figure 2:**
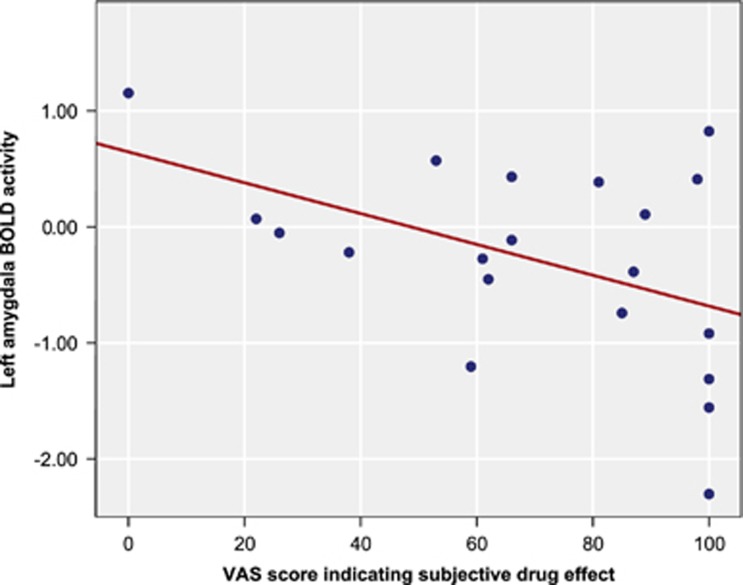
Relation between left amygdala BOLD activity during presentation of fearful faces under the LSD condition and visual analogue scale (VAS) for ‘any subjective drug effects' of LSD (*r*=−0.46, *P*<0.05). BOLD, blood oxygen-level dependent; LSD, lysergic acid diethylamide.

Compared with placebo, administration of LSD reduced neural response to fearful versus neutral faces in the left and right amygdala and the medial frontal gyrus (*P*<0.001, *k*=10; see Figure 1). No increased activity was observed. After correction for multiple comparisons (small volume correction, *P*<0.05 FWE), significantly reduced activity was observed in the left amygdala (MNI_max_ *x*=−15, *y*=9, *z*=−14; cluster size 24; *Z*-score 3.12) and the right medial frontal gyrus (MNI_max_ *x*=15, *y*=42, *z*=16; cluster size 12; *Z*-score 3.78). In addition, there was a significant negative correlation between amygdala blood oxygen-level dependent response to fearful stimuli under the LSD condition and the LSD-induced subjective drug effects (*r*=−0.46, *P*<0.05; see Figure 2).

## Discussion

In summary, the present study used fMRI for we believe the first time to investigate the effects of LSD on the neural substrate of emotional processing. We found that LSD decreased amygdala reactivity to fearful stimuli in healthy subjects. In addition, amygdala deactivation by LSD was associated with its acute subjective psychedelic effects. We administered 100 μg LSD, a representative dose that produces typical and robust psychedelic effects.^19^ In addition, subjects had only had a minimal exposure to recreational drugs and were mostly psychedelic-naive, as is probably the case in patients receiving LSD-assisted psychotherapy.^12^ Our results are consistent with our previously reported findings in a facial emotion recognition task, showing that LSD-impaired recognition of fearful faces compared with placebo.^20^ Our results are also in line with findings obtained after administration of psilocybin, where attenuated recognition of negative facial expressions^21, 22^ and reduced amygdala blood oxygen-level dependent response to fearful faces^23^ were reported. The psilocybin-induced attenuation of amygdala reactivity in response to negative stimuli has consistently been shown to be related to the psilocybin-induced increase in positive mood.^23^

It could be argued that the decreased responsiveness of the amygdala under LSD was due to a drug-induced alteration in visual perception, resulting in the inability to differentiate between the presented facial expressions. However, our results in two doses of LSD (100 μg and 200 μg, respectively) indicated, that LSD specifically impaired recognition of fearful faces, while it did not significantly affect recognition of neutral, happy and angry faces.^20^ Furthermore, subjects in the present study performed well in the gender differentiation task and our whole brain results showed activation in regions typically involved in processing of neutral and fearful faces, respectively.^14^

We observed a significant effect of LSD on the left amygdala. Several studies suggest, that the left amygdala might be particulary involved in processing of negative facial expressions.^24, 25^ It has also been reported that the left amygdala shows lesser habituation to fearful stimuli compared with the right amygdala, which might make it more likely to detect the blood oxygen-level dependent changes in this area.^26, 27^ However, lateralization of the amygdala response is still controversial discussed.^28, 29, 30^ The amygdala receives considerable serotonergic innervation from the raphe nuclei.^31, 32^ LSD mainly acts as an agonist at the serotonin 5-HT_2A_ receptor,^1, 4^ which is expressed in most parts of the amygdala.^33^ Accordingly, this might provide a psychopharmacological basis for the observed effect of LSD on this structure. After administration of selective serotonin reuptake inhibitors in healthy subjects, a decreased amygdala activity in response to negative stimuli was reported.^34, 35^ In depressed patients, amygdala hyperreactivity was resolved after treatment with a selective serotonin reuptake inhibitor.^36, 37^ Those findings indicate that the serotonin system is involved in the modulation of the amygdala response to emotional stimuli.^38^ We also observed decreased activity in the right medial prefrontal cortex (mPFC). The mPFC is anatomically connected to the amygdala^39^ and involved in emotional functions.^40^ Within the mPFC–amygdala circuit, the more ventral parts of the mPFC have been implicated in inhibitory functions,^40^ whereas the more dorsal parts are thought to be part of an ‘aversive-amplification circuit'.^41^ This mechanism has been linked to negative affective bias in anxiety disorder.^42, 43^ Consistent with our findings, serotonin depletion has been shown to increase mPFC activity and functional connectivity between the mPFC and the amygdala in response to fearful stimuli.^44^

The use of psychedelics as an additive in psychotherapy has recently been rediscovered^10, 12, 45^ and our result is relevant for this field of research. Processing biases towards negative stimuli are a feature of several mental diseases, such as depression and social anxiety disorder, and are associated with increased reactivity of the amygdala.^46, 47^ Resolving this processing bias might thus reflect one important and potentially therapeutically useful effect of psychedelic substances by, for example, facilitating the therapeutic alliance^48, 49^ and reducing perception of negative emotions and social cognitive deficits. As we have recently reported, LSD also exhibits some ‘empathogenic' effects (such as increased openness and trust),^2, 20^ which are usually ascribed to substances like 3,4-methylenedioxymethamphetamine (MDMA). The attenuated amygdala reactivity observed in this study is in good accordance with those findings and possibly reflects a neural basis for such effects, which might also be therapeutically beneficial.^49, 50^ However, and in contrast to substances like selective serotonin reuptake inhibitors, the positive long-term effects of psychedelics reported by recent studies^8, 9, 10, 11, 12, 15, 51^ outlast the acute pharmacological effects. It should be further investigated how psychological and biological factors, like neuroplasticity,^52^ contribute to these long-term effects.

Our study has several limitations. First, although the trial was formally double-blinded, assignment to placebo or LSD was unavoidably unblinded by the obvious psychedelic effects caused by the dose used. Second, we did not include in our analyses measures of negative affect. Third, we can only provide data about one moderate dose. Higher doses of psychedelics are possibly difficult to use with fMRI, because they are more likely to induce anxiety,^45^ although the overall effects are still described as positive in the higher doses investigated.^2, 45^ The observed anxiolytic effect probably also depends on personal and environmental factors and might thus be different in the mentally ill or in uncontrolled settings.

## Figures and Tables

**Table 1:** Cumulative lifetime use of legal and illicit drugs of the included subjects

*Nicotine*		*Stimulants*	
No. of subjects with regular use	6/20	No. of subjects who have ever used	4/20
Cigarettes per day (mean/s.d./range)	1.40/4.03/0–10	Lifetime occasions (mean/s.d./range)	0.35/0.5/0–2

*Caffeine*		*Sedatives*	
No. of subjects with regular use	20/20	No. of subjects who have ever used	0/20
Units per day (mean/s.d./range)	3.05/1.96/1–8	Lifetime occasions (mean/s.d./range)	0/0/0

*Alcohol*		*Psychedelics*	
No. of subjects with regular use	20/20	No. of subjects who have ever used	2/20
Units per week (mean/s.d./range)	4.50/2.89/1–10	Lifetime occasions (mean/s.d./range)	0.10/0/0–1

*Cannabis*		*Opioids*	
No. of subjects who have ever used	15/20	No. of subjects who have ever used	1/20
Lifetime occasions (mean/s.d./range)	7.85/13.39/1–50	Lifetime occasions (mean/s.d./range)	0.05/0/0–1

*MDMA*		*Others*	
No. of subjects who have ever used	6/20	No. subjects who have ever used	0/20
Lifetime occasions (mean/s.d./range)	0.60/0.89/0–3	Lifetime occasions (mean/s.d./range)	0/0/0

Abbreviation: MDMA, 3,4-methylenedioxymethamphetamine.
